# Isotherm, kinetic, thermodynamics and reusability data on the adsorption of antidepressant onto silver nanoparticle-loaded biowaste

**DOI:** 10.1016/j.dib.2021.107575

**Published:** 2021-11-13

**Authors:** Kovo G. Akpomie, Jeanet Conradie

**Affiliations:** a Department of Chemistry, University of the Free State, Bloemfontein, South Africa; b Department of Pure and Industrial Chemistry, University of Nigeria, Nsukka, Nigeria

**Keywords:** Nortriptyline, Desorption, Water-treatment, Nanoparticle-based adsorbents

## Abstract

There has been an increase in the use of antidepressant drugs owing to significant economic challenges across the globe. Consequently, the increase in production of such drugs has impacted pharmaceutical pollution of industrial wastewaters discharged into the environment. Hence, there is a need to develop efficient adsorbents for antidepressant wastewater treatment. The impregnation of silver nanoparticles on biowaste was found to be highly effective in the treatment of oil-polluted water but has not been utilized in the adsorption of drugs. Herein, the dataset associated with the adsorption of antidepressants onto *Ananas Comosus* Peel (AP) and Silver nanoparticle-loaded *Ananas Comosus* peel (AgAP) was reported. Batch adsorption methodology was used to study the effect of antidepressant concentration (10–50 mg/L), sonication time (10–120 min), temperature (300–320 K) and adsorbent dosage (0.10–0.18 g). The concentration of antidepressant (Nortriptyline) in solution before and after adsorption was determined by the UV-Spectrophotometer at a maximum wavelength of 239 nm. The isotherm dataset was obtained from the Langmuir, Freundlich, Temkin and Dubinin-Raduskevich models, while kinetic data was evaluated by the pseudo-first order-pseudo-second-order, film diffusion and intraparticle diffusion rate equations. The data on the thermodynamics and adsorbent reusability were also provided. The dataset showed an adsorption capacity of 3.27 mg/g and 4.74 mg/g for AP and AgAP, respectively. The dataset is important to material and environmental scientists and revealed the efficiency of AP and AgAP in the treatment of antidepressant wastewater.

## Specifications Table

**Table:** 

Subject	Chemistry
Specific subject area	Industrial and Physical Chemistry
Type of data	Tables and Figures
How data were acquired	The concentration of the antidepressant in solution before and after adsorption was analyzed by the UV-visible spectrophotometer (Shimadzu UV-1800 model)
Data format	Raw and Analyzed
Parameter for data collection	Effect of drug concentration, sonication time, temperature, adsorbent dosage and adsorbent reusability on adsorption
Description of data collection	The influence of various experimental factors; initial drug concentration (10–50 mg/L), sonication time (10–120 min), temperature (300–320 K), and adsorbent dosage (0.10–0.18 g) on adsorption was performed at optimum conditions (0.1 g adsorbent dosage, 120 min sonication time and 300 K temperature. The isotherm, kinetics, and thermodynamic modeling of the process, as well as the regeneration and reusability of the adsorbent, were analyzed
Data source location	Department of Chemistry, University of the Free State, Bloemfontein, South Africa 29°06′36.1″S 26°11′09.2″E
Data accessibility	The data sets are deposited on Mendeley Data https://doi.org/10.17632/pk98wm9j52.1

## Value of the Data

• The dataset is essential to elucidate how the impregnation of silver nanoparticles on biowaste impacts the adsorption capacity for antidepressants sequestrated from contaminated water.
• Dataset provides valuable information on how variations in temperature, time, drug concentration, and material dosage influence the uptake of antidepressants onto nanoparticles-loaded biowaste.
• The data on the isotherm, kinetics, and thermodynamics can be used by Chemical Engineers for the design of adsorption systems in the treatment of drug contaminated waters.
• The data can be used by Environmental scientists for comparison of the adsorption performance of the material with newly synthesized adsorbents.
• The dataset helps in fostering the development of efficient adsorbent materials for the disinfection of antidepressant drug polluted waters to abate the increasing pollution of environmental waters.
• The data also provides significant insights into the reuse of silver nanoparticles-loaded biowaste for the adsorption of drugs as an eco-friendly material to preserve water quality.

## 1 Data Description

**Fig. 1:**
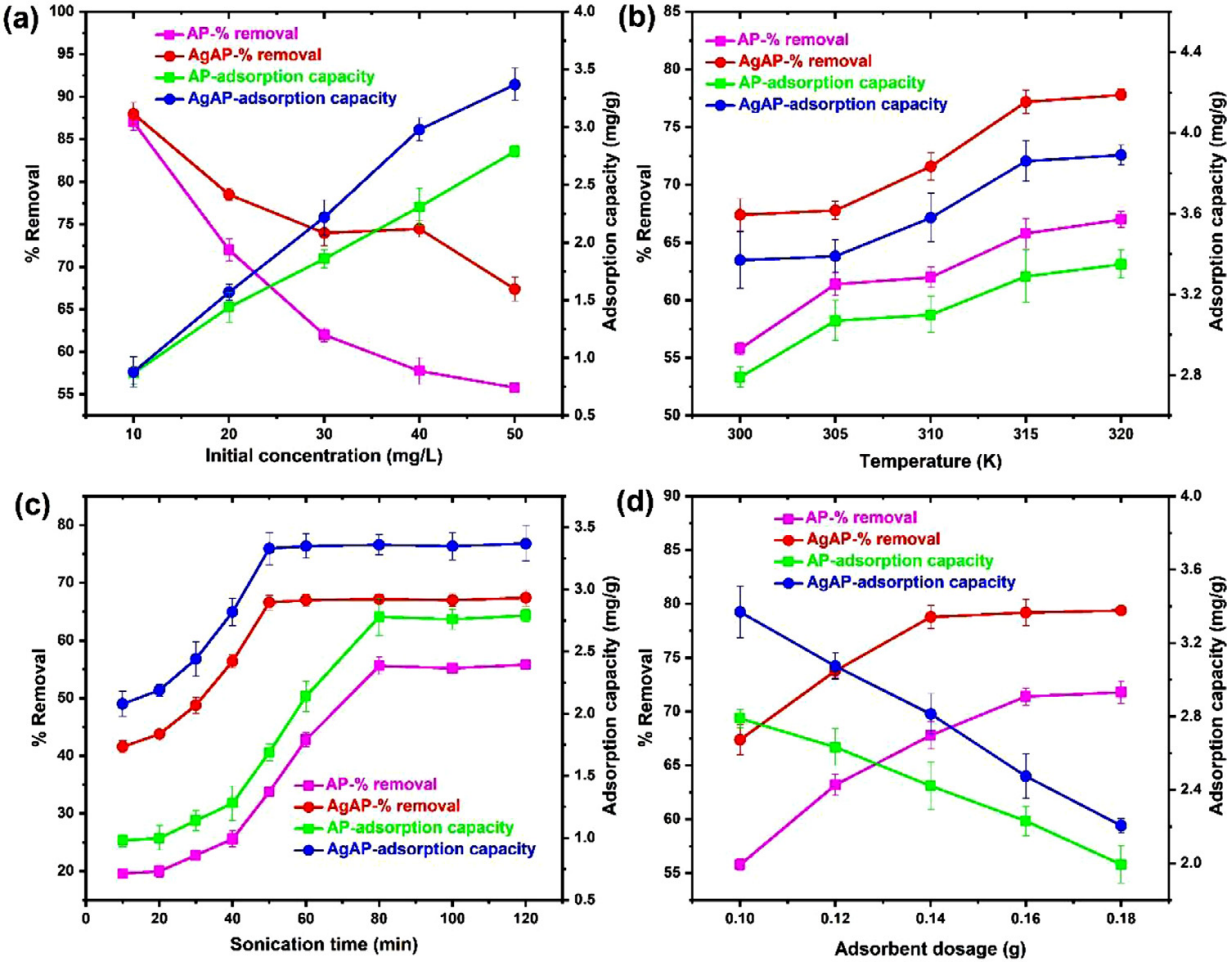
The influence of (a) initial drug concentration (pH 7.0, sonication time 120 min, temperature 300 K), (b) temperature (drug concentration 50 mg/L, pH 7.0, sonication time 120 min), (c) sonication time (drug concentration 50 mg/L, pH 7.0, temperature 300 K) and (d) adsorbent dosage (pH 7.0, drug concentration 50 mg/L, temperature 300 K, sonication time 120 min) for the adsorption of antidepressant drug onto biowaste and silver nanoparticles-loaded biowaste.

**Fig. 2:**
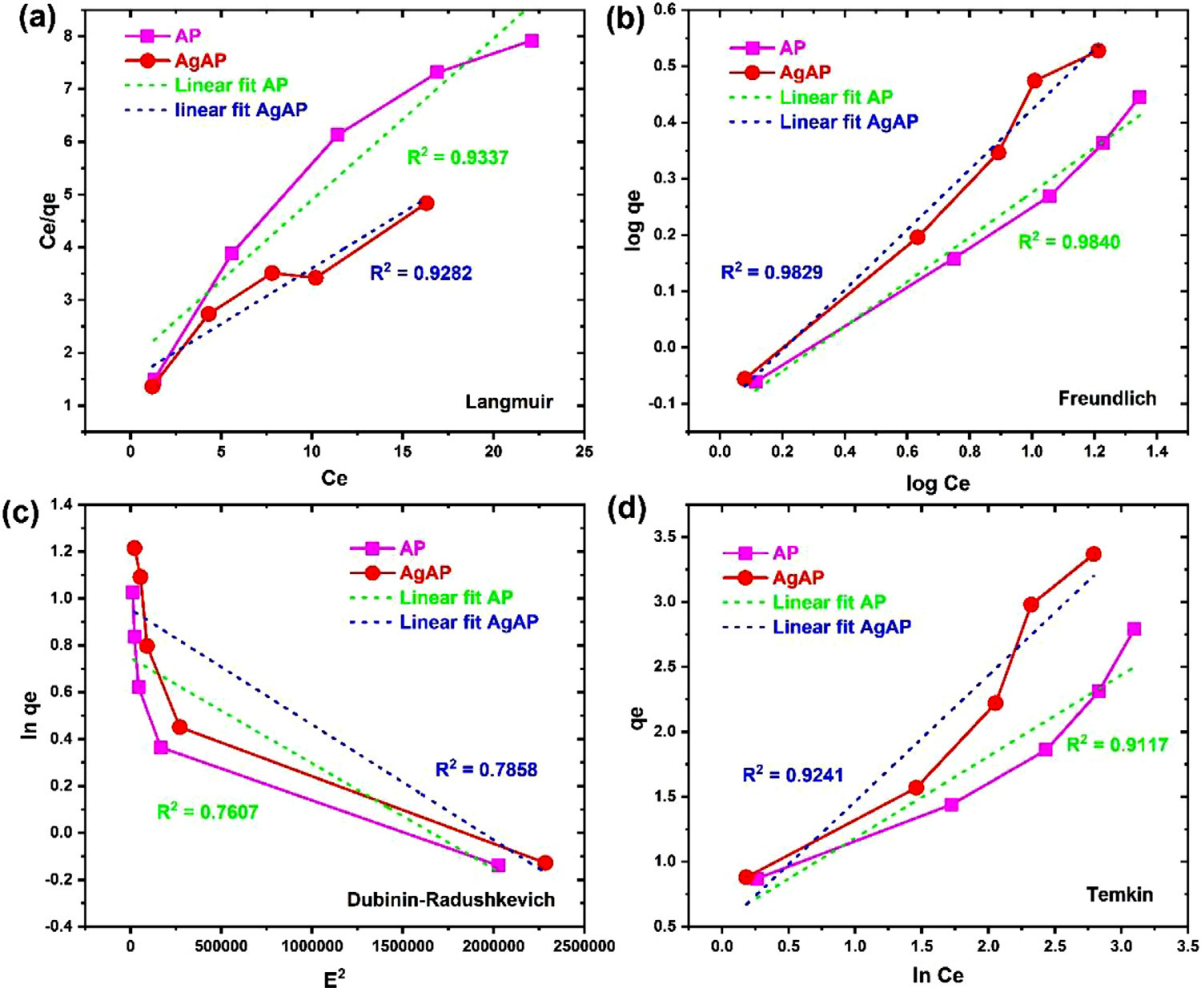
The (a) Langmuir (b) Freundlich (d) Dubinin-Radushkevich and (d) Temkin isotherm models plots for the adsorption of antidepressant drug onto biowaste and silver nanoparticles-loaded biowaste.

**Fig. 3:**
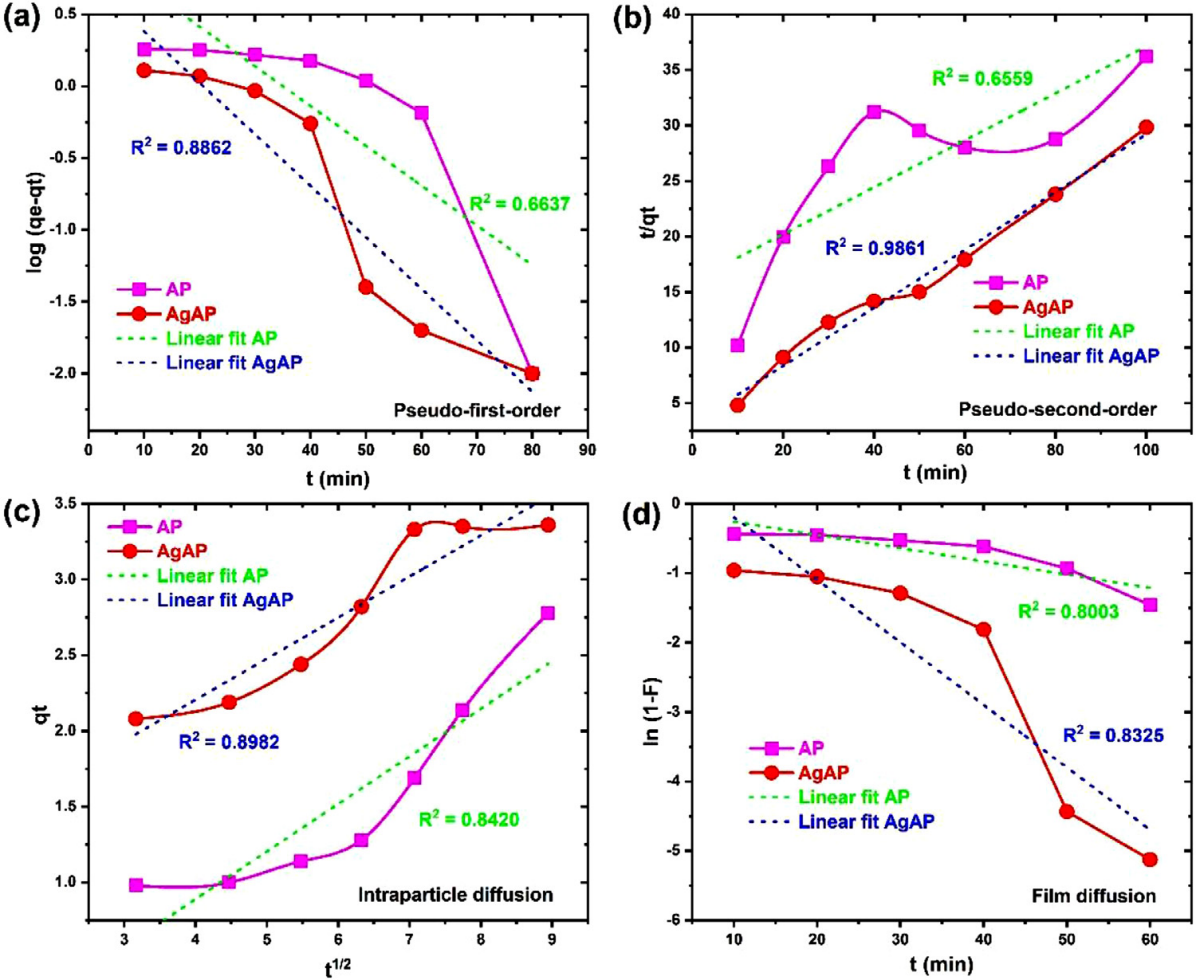
The (a) Pseudo-first-order (b) Pseudo-second-order (c) intraparticle diffusion and (d) film diffusion model plots for the adsorption of antidepressant onto biowaste and silver nanoparticles-loaded biowaste.

**Fig. 4:**
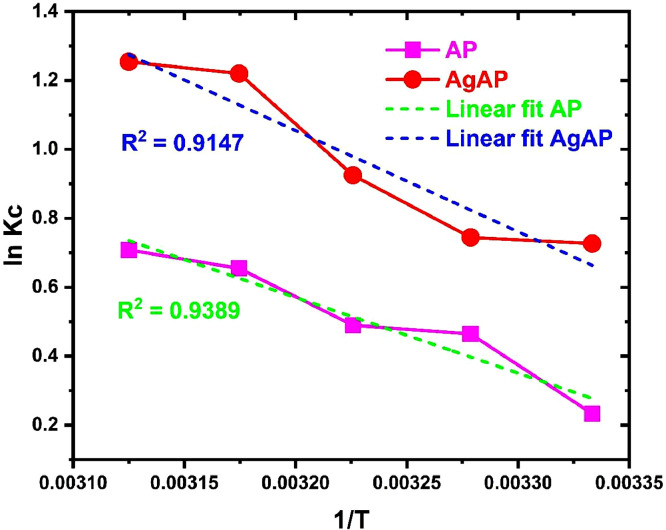
The Van't Hoff thermodynamic plot for the adsorption of antidepressant onto biowaste and silver nanoparticles-loaded biowaste.

**Fig. 5:**
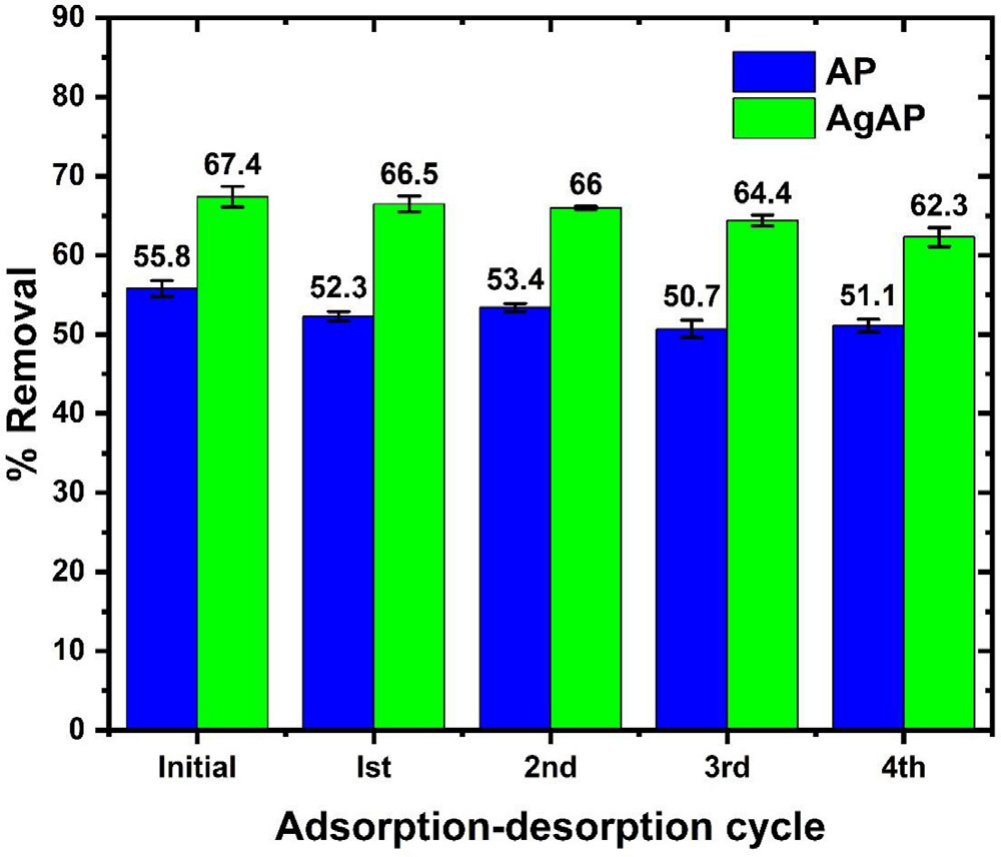
The regeneration and reuse of the biowaste and silver nanoparticles-loaded biowaste for the adsorption of antidepressant.

The data on the adsorption of antidepressants from contaminated solution onto *Ananas comosus* peel (AP) and silver nanoparticles-loaded *Ananas Comosus* peel (AgAP) are reported in this article. The effects of initial drug concentration, temperature, sonication time and adsorbent dose on drug adsorption are presented in Fig. 1. The Langmuir, Freundlich, Temkin and Dubinin-Radushkevich model plots used to analyze equilibrium isotherm parameters are illustrated in Fig. 2, while the obtained isotherm parameters are presented in Table 1. Moreover, the Pseudo-first-order, Pseudo-second-order, intraparticle diffusion and film diffusion kinetic model plots for the adsorption process are illustrated in Fig. 3, while the calculated kinetic parameters are presented in Table 2. In addition, the Van't Hoffs thermodynamic plot for the adsorption of the antidepressant onto AP and AgAP is shown in Fig. 4 and the thermodynamic parameters obtained are presented in Table 3. The reusability of AP and AgAP for drug adsorption is illustrated in Fig. 5. All related primary data are deposited on Mendeley Data (DOI: 10.17632/pk98wm9j52.1). The raw data associated with the effect of adsorbent dose on adsorption is presented (Dataset 1), while Dataset 2 is the raw data for the effect of concentration and isotherm of the adsorption process. The raw data associated with the influence of time and kinetics is provided in Dataset 3. In addition, Dataset 4 is the raw data associated with the effect of temperature and thermodynamics on antidepressant adsorption onto AP and AgAP.

**Table 1:** Isotherm parameters for the adsorption of antidepressant onto biowaste and silver nanoparticles-loaded biowaste.

Model	Parameter	AP	AgAP
Langmuir			
	K_L_ (L/mg)	0.166	0.141
	q_L_ (mg/g)	3.270	4.741
	R^2^	0.9337	0.9282
	SSE	1.863	0.459
Freundlich			
	N	2.520	1.872
	K_F_ (L/g)	0.757	0.774
	R^2^	0.9840	0.9829
	SSE	0.002	0.004
Temkin			
	B (mg/g)	0.628	0.969
	A (L/g)	2.423	1.668
	R^2^	0.9117	0.9241
	SSE	0.196	0.312
Dubinin-Radushkevich			
	qm (mg/g)	2.108	2.594
	(mol^2^/J^2^)	4.494 × 10^−7^	4.912 × 10^−7^
	R^2^	0.7607	0.7858
	SSE	0.197	0.252

**Table 2:** Kinetic parameters for the adsorption of antidepressant onto biowaste and silver nanoparticles-loaded biowaste.

Model	Parameter	AP	AgAP
	qe_exp_ (mg/g)	2.790	3.370
Pseudo-first-order			
	K_1_ (min^−1^)	0.064	0.083
	qe_cal_ (mg/g)	9.337	5.515
	R^2^	0.6637	0.8862
Pseudo-second-order			
	K_2_ (mg/g min)	2.793 × 10^−3^	21.42 × 10^−3^
	qe_cal_ (mg/g)	4.731	3.837
	R^2^	0.6559	0.9861
Intraparticle diffusion			
	K_d_ (mg/g min^−1/2^)	0.314	0.271
	C	0.366	1.122
	R^2^	0.8420	0.8982
Liquid film diffusion			
	K_fd_	0.019	0.090
	Y	0.067	0.706
	R^2^	0.8003	0.8325

**Table 3:** Thermodynamic parameters for the adsorption of antidepressant onto biowaste and silver nanoparticles-loaded biowaste.

Sorbent	T(K)	ΔG^o^ (kJ/mol)	ΔH^o^ (kJ/mol)	ΔS^o^ (J/mol K)	R^2^
AP					
	300	‒0.581	18.258	63.166	0.9389
	305	‒1.177			
	310	‒1.262			
	315	‒1.714			
	320	‒1.884			
AgAP					
	300	‒1.812	24.386	86.801	0.9147
	305	‒1.888			
	310	‒2.383			
	315	‒3.194			
	320	‒3.336

## 2 Experimental Design, Materials and Methods

### 2.1 Materials preparation

The Pineapple (*Ananas Comosus*) biowaste was obtained from Checkers, Mimosa mall, Bloemfontein, South Africa. The preparation and characterization of the *Ananas Comosus* peel (AP) biowaste adsorbent and silver nanoparticles-loaded *Ananas Comosus* peel (AgAP) adsorbent have been described in our previous work [1]. In this preparation, the pineapple peels were removed manually with a knife and washed with tab water to remove surface impurities. The washed peels were then cut into smaller sizes, then sundried for 48 h, followed by oven drying at 80 °C for 2 h in a laboratory oven (Labcon model). The dried peels were powdered using the mortar and pestle, followed by the addition of 200 mL of 0.1 M nitic acid to 40 g of the powder. The mixture was stirred for 1 h, then washed with water until pH 7.0, followed by oven-drying at 70 °C for 24 h. The sample was then pulverized and passed through a 100-µm mesh screen to obtain the AP adsorbent. The silver nanoparticle-loaded *Ananas Comosus* (AgAP) was synthesized by contacting 10.0 g of AP (without nitric acid treatment) with 100 mL of silver nitrate solution contained in a beaker on a hotplate. The silver nitrate solution was initially stirred at 30 °C for 30 min with a magnetic stirrer prior to the addition of the AP. The mixture was stirred for 2 h, followed by the addition of 3 mL (1% trisodium citrate) with further stirring for 8 h. Thereafter, the mixture was left to settle for 30 min, then centrifuged at 8000 rpm for 1 h. This was followed by washing with excess water until pH 7.0, centrifuging and then oven drying at 70 °C for 24 h. The powder was then pulverized and passed through a 100-μmmesh screen to obtain the as-prepared AgAP adsorbent.

### 2.2 Antidepressant adsorption, desorption, and reusability

The antidepressant (Nortriptyline) was obtained in its hydrochloric form (Nortriptyline hydrochloride) from Sigma Aldrich, South Africa, and used without further purification. The adsorption of the antidepressant onto AP and AgAP was performed by batch adsorption technique. In a typical procedure, 100 mg/L stock solution of the antidepressant was prepared followed by serial dilutions of the stock to concentrations of 10–50 mg/L. Thereafter, 0.10 g of adsorbent was added to 10 mL of 50 mg/L antidepressant solutions at pH 7.0 and ultra-sonicated for 120 min at a room temperature of 300 K, then filtered. The concentration of antidepressants remaining in solution after adsorption was then determined by the UV-Spectrophotometer (Shimadzu UV-1800 model) at a maximum wavelength of 239 nm. The effect of adsorbent dosage (0.10–0.18 g), sonication time (10–180 min) and temperature (300–320 K) was also studied by varying the factor of interest while keeping the others constant at the optimum conditions. The percentage removal and adsorption capacity (mg/g) of the materials were calculated respectively [2]:

(1) $$\%Removal\;=\left(\frac{\left(C_{o}-C_{e}\right)}{C_{o}}\right)*100$$

(2) $$qe=\frac{\left(C_{o}-C_{e}\right)V}{m}$$

Where *C_o_* and *C_e_* in mg/L correspond to the initial and equilibrium antidepressant concentrations in solution, respectively, *V* (L) is the volume of solution used and *m* (g) is the mass of AP and AgAP used in adsorption.

The desorption experiment was carried out by contacting the antidepressant-loaded adsorbents with 15 mL of ethanol with constant stirring for 10 min. Thereafter the solution was left to settle and the ethanol decanted before drying the regenerated adsorbents in the oven at 100 °C for 3 h. The regenerated AP and AgAP were then reused for antidepressant adsorption and four adsorption-desorption cycles were performed to ascertain the reusability. Each adsorption and desorption experiment was performed twice and the mean value was computed for quality assurance.

### 2.3 Isotherm modeling

The isotherm modeling of antidepressant adsorption onto AP and AgAP was evaluated using four adsorption isotherms, namely; Langmuir, Freundlich, Temkin and Dubinin-Radushkevich models given respectively [3,4]:

(3) $$\frac{C_{e}}{q_{e}}=\frac{1}{q_{L}K_{L}}+\frac{C_{e}}{q_{L}}$$

(4) $$\log q_{e}=\;\log K_{F}+\;\left(\frac{1}{n}\right)\log C_{e}$$

(5) $$q_{e}=B\ln A+B\ln C_{e}$$

(6) $$\text{ln}q_{e}=\text{ln}q_{m}+\beta \varepsilon 2$$

Where C_e_ (mg/L) and q_e_ (mg/g) corresponds to the equilibrium dye concentration and adsorption capacity, respectively. K_L_ (L/mg) is the Langmuir constant and q_L_ (mg/g) is ascribed to the maximum monolayer capacity. The n and K_F_ (L/g) are Freundlich constants corresponding to the adsorption intensity and uptake capacity, respectively, The constants q_m_ (mg/g) and *β* (mol^2^/J^2^) are the Dubinin-Radushkevich constants and ε is the Polanyi potential, while *B* and *A* (L/mg) corresponds to the Temkin heat of adsorption and binding energy, respectively.

### 2.4 Kinetic modeling

The kinetics of antidepressant uptake onto AP and AgAP were evaluated by four kinetic models, namely, Pseudo-first-order (PF), Pseudo-second-order (PS), Intraparticle diffusion (ID) and film diffusion (FD) models equations, given respectively [5], [6], [7]:

(7) $$\log\left(q_{e}-q_{t}\right)=\log q_{e}-\frac{K_{1}}{2.303}t$$

(8) $$\frac{t}{q_{t}}=\frac{1}{K_{2}{q_{e}}^{2}}+\frac{t}{q_{e}}$$

(9) $$q_{t}=K_{d}t^{1\;/\;2}+C$$

(10) $$ln\left(1-F\right)=Y-K_{FD}t$$

Where q_t_ (mg/g) is the adsorption capacity at time t (min), K_I,_ K_2,_ K_d,_ and K_FD_ corresponds to the rate constants of the PF, PS, ID, and FD kinetic models, respectively. Y and C are the intercepts of the FD and ID models, while F is the fractional equilibrium attainment.

### 2.5 Adsorption thermodynamics

The thermodynamic parameters for the uptake of antidepressants onto AP and AgAP were elucidated from the Gibbs free energy and Van't Hoff equations, given respectively as [8,9]:

(11) $$\Delta G^{0}=\;-RT\;\ln K_{c}$$

(12) $$\ln K_{C}=-\left(\frac{\Delta H^{0}}{RT}\right)+\left(\frac{\Delta S^{0}}{R}\right)$$

Where ∆G°, ∆H° and ∆S° correspond to the changes in free energy, enthalpy and entropy, respectively, R (8.314 J/mol K) is the universal gas constant, K_c_ is the equilibrium constant and T (K) is the absolute temperature.

## Ethics Statements

Not applicable.

## CRediT authorship contribution statement

**Kovo G. Akpomie:** Conceptualization, Investigation, Formal analysis, Writing – review & editing. **Jeanet Conradie:** Writing – review & editing.

## Declaration of Competing Interest

The authors declare that they have no known competing financial interests or personal relationships which have or could be perceived to have influenced the work reported in this article.
